# Radiological Assessment of Coronal Plane Alignment of the Knee Phenotypes in the Romanian Population

**DOI:** 10.3390/jcm13144223

**Published:** 2024-07-19

**Authors:** Serban Dragosloveanu, Bogdan-Sorin Capitanu, Radu Josanu, Diana Vulpe, Romica Cergan, Cristian Scheau

**Affiliations:** 1 Department of Orthopaedics and Traumatology, The “Carol Davila” University of Medicine and Pharmacy, 050474 Bucharest, Romania; 2 Department of Orthopaedics, “Foisor” Clinical Hospital of Orthopaedics, Traumatology and Osteoarticular TB, 021382 Bucharest, Romania; 3 Department of Anatomy, The “Carol Davila” University of Medicine and Pharmacy, 050474 Bucharest, Romania; 4 Department of Radiology and Medical Imaging, “Foisor” Clinical Hospital of Orthopaedics, Traumatology and Osteoarticular TB, 021382 Bucharest, Romania; 5 Department of Physiology, The “Carol Davila” University of Medicine and Pharmacy, 050474 Bucharest, Romania

**Keywords:** coronal plane alignment of the knee, CPAK, phenotype, Romanian population, morphometry, radiology, anatomy, pathophysiology

## Abstract

**Background**: The Coronal Plane Alignment of the Knee (CPAK) classification system has been developed as a comprehensive framework delineating nine coronal plane phenotypes, based on arithmetic hip–knee angle (aHKA) and joint line obliquity (JLO). Our study aimed to assess the prevalence of knee phenotypes in the Romanian population using the CPAK classification, encompassing both osteoarthritic and healthy cohorts. **Methods**: We conducted an observational cross-sectional study, analyzing data from 500 knees with osteoarthritis and 500 healthy knees that met the inclusion criteria. Demographic data were collected, and radiological parameters including lateral distal femoral angle (LDFA), medial proximal tibial angle (MPTA), aHKA, and JLO were measured. Knee phenotypes were categorized using the CPAK classification. **Results**: In the osteoarthritic cohort, the most prevalent CPAK phenotype was type I (42.4%), characterized by varus alignment and an apex distal joint. Conversely, in the healthy population, CPAK type II, indicating neutral alignment and an apex distal joint, was the most prevalent phenotype (39.0%). CPAK types VII, VIII, and IX were rare. **Conclusions**: Our findings demonstrate similarities in knee phenotypes compared to other populations, with some minor differences and particularities. The CPAK classification proves to be a valuable tool in assessing knee tyalignment.

## 1. Introduction

Osteoarthritis (OA) of the knee stands as the prevalent articular ailment and is progressively becoming a leading cause of disability among the elderly population, attributed mainly to heightened life expectancy and obesity [1,2]. Total knee arthroplasty (TKA) has emerged as one of the most economically viable and efficacious surgical interventions in orthopedics, offering dependable outcomes for patients. Notwithstanding the commendable success rates associated with TKA, attaining a substantial proportion of satisfied patients remains arduous, with 20% of individuals expressing dissatisfaction [3].

The mechanical alignment (MA) technique has been widely adopted in TKA since its inception. [4]. The principal objective of MA is to achieve a neutral mechanical axis of the limb [5,6]. Although MA offers several advantages, it is important to recognize its limitations in addressing “pre-arthritic” knee alignment. This is particularly significant considering that only a small subset of the population exhibits a naturally neutral knee alignment; therefore, soft tissue release is often deemed necessary [7]. The objective of anatomical alignment is to achieve a knee that maintains neutral alignment, ideally with a joint line positioned 2–3 degrees in varus relative to the mechanical axis. While initial outcomes are often favorable, concerns have arisen regarding premature polyethylene wear [6,8]. To address these drawbacks, Kinematic Alignment (KA) was advocated by Howell et al. [9]. KA is geared toward restoring the intrinsic alignment of the knee while minimizing the need for soft tissue release and enhancing ligament balance [10]. However, achieving accurate replication of native anatomy through KA techniques is challenging without prior knowledge of an individual’s constitutional alignment. This complexity is compounded by the tendency for native alignment to deteriorate as arthritis progresses, with the prediction of the original native alignment becoming difficult [11].

The Coronal Plane Alignment of the Knee (CPAK) classification was first described by Macdessi et al. in 2021 and divides coronal alignment into nine phenotypes, determined by the relationship between the arithmetic Hip Knee Angle (aHKA) and Joint Line Obliquity (JLO), derived from the measurements of the Medial Proximal Tibial Angle (MPTA) and Lateral Distal Femoral Angle (LDFA) [12]. This classification system proves valuable in anticipating the constitutional alignment among osteoarthritic patients and may serve as a predictive tool to identify those individuals who would derive the greatest advantage from the restoration of their constitutional alignment [13]. Although widely employed in numerous studies and clinical contexts, ongoing research endeavors aim to further enhance and corroborate its applicability in clinical settings. Pagan et al. conducted a systematic literature review concerning the distributions of knee phenotypes based on the CPAK classification. Their findings indicated considerable variability in CPAK distributions across different countries [14]. Consequently, it is deemed crucial to ascertain the CPAK phenotype for a particular population, considering the multiple factors that can impact knee alignment. To the best of our knowledge, no studies have explored the CPAK phenotype in the Romanian population to date.

In this study, we aimed to assess the prevalence of knee CPAK phenotypes in the Romanian population, both healthy and arthritic, in a dedicated orthopedics center. We hypothesized that there is a different distribution of the CPAK phenotypes among the two groups. Additionally, we will assess our results in comparison with other European and Asian cohorts.

## 2. Materials and Methods

### 2.1. Study Design

Our observational cross-sectional study included 1000 patients from the database of the “Foisor” Clinical Hospital of Orthopaedics, Traumatology, and Osteoarticular Tuberculosis, between January 2016 and December 2023, divided into two cohorts: the healthy knee cohort (500 patients) and the osteoarthritic knee cohort (500 patients).

The healthy knee group consisted of consecutive patients who presented at our clinic for anterior cruciate ligament reconstruction or partial meniscectomy via arthroscopic procedures and who underwent digital full-length standing anteroposterior view X-rays. To mitigate the potential influence of associated pathologies on the study, we focused our examination on the unaffected limb, without any complaint-related or lower limb deformity.

The osteoarthritic knee group consisted of consecutive adults scheduled for primary TKA in our clinic, with complaints suggestive of OA and radiographic evidence of Kellgren–Lawrence grade 3/grade 4. We examined the limb proposed for surgery on the digital full-length standing anteroposterior view X-rays like in the healthy group.

Patients with a history of surgical intervention involving the lower limbs, as well as those with a history of or known cases of skeletal tuberculosis or any other lower limb pathology identified on radiographic imaging, were excluded from the study. Furthermore, patients with incomplete data or radiographs that were of suboptimal quality were also omitted from the analysis.

Ethics approval was obtained from the Ethics Council of “Foisor” Clinical Hospital of Orthopaedics, Traumatology, and Osteoarticular TB with registration number 1160/31.01.2024. Written informed consent was obtained from all patients included in the study. The study followed the ethical principles for medical research stipulated by the Declaration of Helsinki in 1964 and its later amendments.

### 2.2. Image Acquisition and Analysis

All participants enrolled in this investigation provided digital full-length standing antero-posterior view X-rays at our Department of Radiology and Medical Imaging. The X-ray examinations were conducted using a DigitalDiagnost R3.1 machine (Philips Medical Systems Nederland B.V, Amsterdam, The Netherlands). Images were obtained using a focus-film distance of 240 cm. The beam was centered on the popliteal fold, with a centered patella. The beam was horizontal and orthogonal to the limbs, and an anti-diffusion grid was used. The images were obtained on the wall stand using three-step exposures and post-processing stitching was automatically performed. The electrical parameters employed were 85 kV and 250 mA.

Subsequently, the radiographic images stored in the hospital’s archiving and communication system (PACS) were analyzed for each participant using mediCAD^®^ 2D Version 7.0 (mediCAD Hectec GmbH, Altdorf/Landshut, Germany), specialized imaging software designed for orthopedic applications.

### 2.3. Morphometric Radiographic Parameters

**Figure 1:**
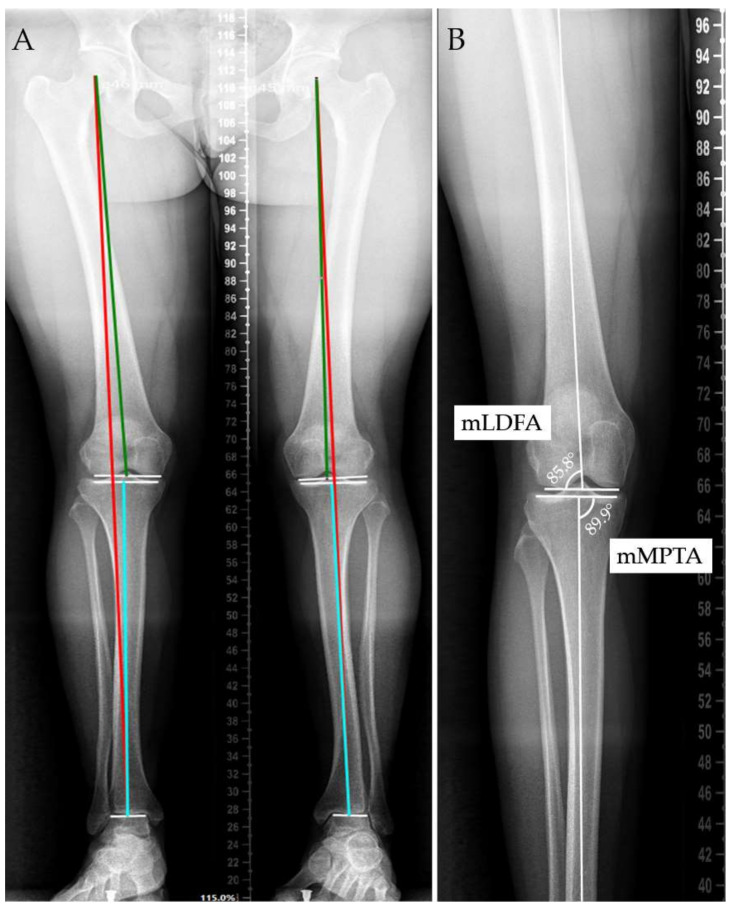
Method of measurement for the radiological parameters of the study. (**A**) Physiological axes of the lower limb; teal line=mechanical tibial axis, green line=mechanical femoral axis, red line=lower limb mechanical axis; (**B**) mLDFA (mechanical lateral distal femoral angle) and mMPTA (mechanical medial proximal tibial angle).

Measurements were conducted by two observers in both cohorts, employing identical criteria and using the same imaging software in order to minimize measurement bias. The reliability of measurements was evaluated using Cohen’s Kappa coefficient on a sample of 50 knees from the OA cohort and 50 knees from the healthy cohort. The following measurements were utilized, as illustrated in the image below (Figure 1A): the center of the femoral head, the center of the ankle joint, the mechanical tibial axis (teal line), the mechanical femoral axis (green line), and the lower limb mechanical axis (i.e., the Mikulicz axis, red line) [15].

For the subsequent measurements, we adhered to the parameters delineated in the original depiction of CPAK classification by MacDessi et al. to accurately replicate the methodology [12]. The mechanical lateral distal femoral angle (mLDFA) is defined as the angle formed between the femoral mechanical axis and the joint line of the distal femur, with a normal range of 87 ± 3 degrees. Similarly, the mechanical medial proximal tibial angle (mMPTA) is characterized as the angle formed between the tibial mechanical axis and the joint line of the proximal tibia, with a normal range of 87 ± 3 degrees (Figure 1B) [12].

The arithmetic hip–knee ankle angle (aHKA) denotes the disparity between mMPTA and mLDFA and serves as a measure for assessing constitutional alignment. The optimal range for Neutral aHKA is within 0 degrees, with a permissible deviation of plus or minus 2 degrees. A value less than −2 signifies a varus alignment, while a value exceeding +2 indicates a valgus alignment [12].

Conversely, joint line obliquity (JLO) is determined by the summation of the mMPTA and mLDFA angles, operating independently from the mechanical axis of the lower limb. JLO delineates the orientation of the joint line relative to the floor during double-leg stance. When the sum of these two angles equals 180 degrees with a permissible deviation of plus or minus 2 degrees, the joint line is considered neutral. A sum exceeding 183 degrees suggests an apex proximal joint line, whereas a sum less than 177 degrees indicates an apex distal joint line [12].

The CPAK classification integrates the two distinct variables of aHKA and JLO. By juxtaposing the three subgroups of aHKA with the three subgroups of JLO within a matrix, the CPAK classification yields nine distinct phenotypes of knee alignment, as described by MacDessi et al. All data utilized for this manuscript are outlined in Table 1 [12].

**Figure 2:**
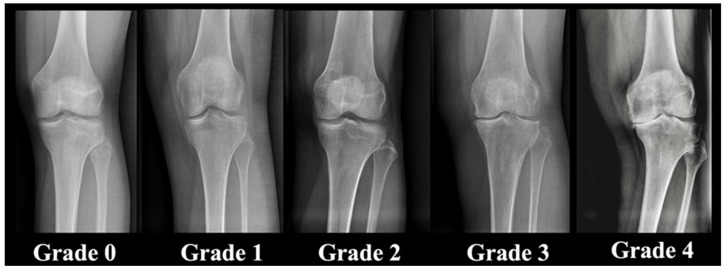
Kellgren–Lawrence classification exemplified on 5 patients from the patient cohorts [16].

We utilized the Kellgren–Lawrence score to categorize the severity of knee osteoarthritis as it is the predominant method employed in our clinical practice [16] (Figure 2).

### 2.4. Statistical Analysis

We estimated the sample size by committing to a 0.05 significance level and a 95% statistical power with an equal enrollment ratio; based on previous studies, we estimated a 1-degree difference ± 3 degrees standard deviation of the studied parameters between the study groups. We collected a series of demographic parameters such as gender, age, and BMI. Data distribution was tested for normality using the Shapiro–Wilk test. Student’s t-test was used to compare continuous variables such as age, BMI, or morphometric parameters across patient subgroups. The chi-squared test was employed to determine whether the frequency of one parameter, such as gender was significantly higher in a specific group. Fischer’s test was used to determine if there are nonrandom associations between two categorical variables. To compare the means of two independent groups when the assumption of equal variances is not respected, we employed Welch’s test. We used multiple observers to mitigate observer bias. The reliability of measurements was evaluated using Cohen’s Kappa coefficient. The statistical software used in this study was SPSS^®^ Statistics Version 26, 64-bit edition (IBM, Armonk, NY, USA). Individual results were considered statistically significant for *p*-values < 0.05. Due to the performance of multiple comparisons between the healthy and osteoarthritic groups, as well as among various subgroups, we implemented Bonferroni correction in order to prevent the risk of type I errors associated with multiple testing. Specifically, we performed a total of 10 comparisons, therefore, we considered the significance level of each individual test of 0.005. By applying this criterion, we aimed to decrease the rate of false-positive findings and to enhance the validity of our results. We performed a sensitivity analysis to ensure the reliability of our findings and the robustness of the reported data by varying key parameters such as measurement variables and sample size. The sensitivity analysis showed the effect of variability in the measurement of the radiographic parameters and confirmed the consistency of the reported results throughout variable scenarios. Also, sample size variations significantly impacted the statistical power, emphasizing the relevance of our selected cohort sizes in the adequate identification of significant differences.

## 3. Results

The analysis encompassed a total of 1000 patients, with cohort 1 (arthritic population) comprising 500 knees and cohort 2 (healthy population) comprising 500 knees. Table 2 provides an overview of the study population characteristics.

### 3.1. Knee Alignment

**Figure 3:**
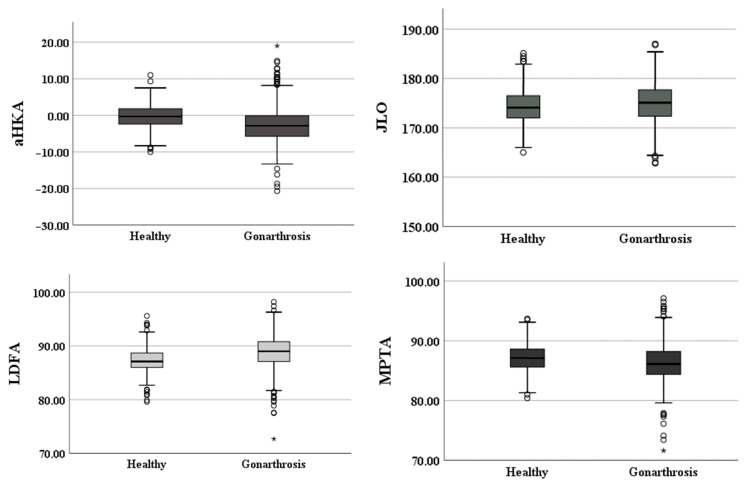
Comparative graphical view of the differences and distributions of the measured angles and distances in the two study cohorts; circle=outlier; asterisk=extreme outlier.

The alignment of the knee was assessed by utilizing aHKA. In the osteoarthritic group, the mean aHKA was recorded as −2.59 ± 5.21 degrees, indicating a predominant varus alignment within this cohort. Stratified by gender, females exhibited a mean aHKA of −2.02 ± 5.31 degrees, whereas males demonstrated a mean aHKA of −4.28 ± 4.51 degrees, with the gender differences being statistically significant (*p* < 0.001) (Figure 3).

Conversely, in the healthy group, the mean aHKA was determined to be −0.18 ± 3.13 degrees, indicative of a neutral alignment falling within the normal range of −2 to +2 degrees. When segmented by gender, females displayed a mean aHKA of −0.93 ± 2.93 degrees, while males exhibited a significantly different mean value of −0.75 ± 3.08 degrees (*p* < 0.001) (Figure 3).

### 3.2. Joint Line Obliquity

In both study cohorts, the apex distal type predominated. Within the osteoarthritic group, the mean JLO was 174.32 ± 3.24, whereas in the healthy group, it was 174.96 ± 4.16 (Figure 3). Stratified by gender, in the healthy cohort JLO averaged 174.75 ± 3.55 in females and 174.10 ± 3.05 in males (*p* = 0.032).

### 3.3. CPAK Classification

**Figure 4:**
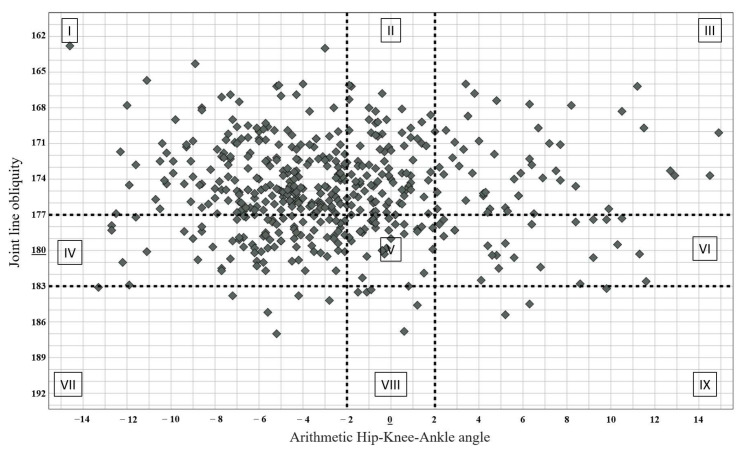
CPAK phenotype distribution in the osteoarthritic cohort.

**Figure 5:**
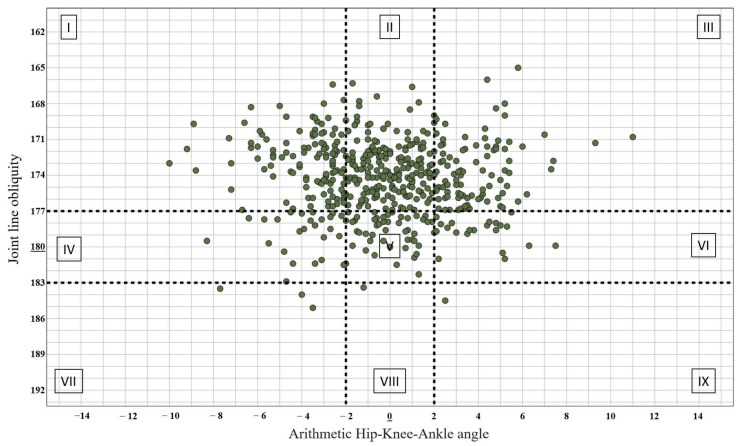
CPAK phenotype distribution in the healthy cohort.

Cohort distribution by CPAK phenotype is summarized in Figure 4 and Figure 5, while gender stratification is further provided in Table 3. Within cohort 1 comprising osteoarthritic patients, the majority of individuals were categorized as type I (*n* = 201, 41.88%), with type II (*n* = 83, 17.29%) being the next most prevalent. type IX (*n* = 3, 0.63%) and type VIII (*n* = 5, 1.04%) were infrequent within this cohort. In cohort 2 comprising healthy patients, the majority were classified as type II (*n* = 195, 37.60%), with type 1 (*n* = 112, 21.60%) following closely. type IX (*n* = 1, 0.2%) and type VIII (*n* = 1, 0.2%) were infrequently observed within this group (Table 3).

Stratified by gender, within the osteoarthritic group, CPAK type I (51.64%) and type IV (18.03%) were the most prevalent among males, whereas among females, CPAK type I (38.55%), type II (19.27%), and type IV (14.80%) were the most commonly observed (Table 2). However, within the healthy group, CPAK type II (41.12%) and type I (28.79%) were predominantly observed among males, while among females, CPAK type II (32.94%), type III (28.24%), and type V (15.88%) were the most frequently encountered.

### 3.4. Reliability

Intraobserver correlation assessment revealed Kappa values of 0.889 for mLDFA and 0.946 for mMPTA. Interobserver agreement was 0.947 for mLDFA and 0.912 for mMPTA.

## 4. Discussion

The principal aim of this investigation was to assess the knee phenotype among both healthy and arthritic individuals within the Romanian population by utilizing the CPAK classification. Our findings revealed that within the arthritic cohort, CPAK type I, characterized by varus alignment and an apex distal joint, was the predominant phenotype (42.4%). Conversely, in the healthy population, CPAK type II, denoting neutral alignment and an apex distal joint, was the most prevalent phenotype (39%).

These findings align closely with results observed in previous investigations examining the distribution of CPAK phenotypes within diverse populations. In cohorts with arthritic knees, CPAK type I emerged as the predominant phenotype in studies conducted in France (33.4%), India (33.6%), Portugal (23%), and Japan (53.8%), while CPAK type II predominated in Australia (32.8%) and Turkey (31.6%). Conversely, among cohorts with healthy knees, CPAK type II was the most prevalent phenotype in Belgium (39.2%), Taiwan (39.3%), Turkey (41.5%), Portugal (42%), and India (25.6%) [14,17,18,19].

Contrasting outcomes emerged from a cross-sectional investigation conducted by Coetzee et al. in the South African population. Their study indicated that CPAK type III (characterized by valgus alignment and an apex distal joint) was the predominant subtype (28.6%) observed among arthritic subjects. They posit that their results may be influenced by the notable proportion of female participants in their study, accounting for 77.9% [20]. In our investigation, type III within the arthritic cohort comprises only 9.8%, while in the female subset, CPAK type III accounts for 10.93%.

In a recent investigation conducted by Hubert et al., a comprehensive analysis of 8739 preoperative long leg radiographs of patients scheduled for TKA was undertaken to delineate gender-specific patterns of knee morphology according to the CPAK classification. The study revealed a higher prevalence of varus alignment in the male population, while valgus or neutral alignment was more frequently observed in females. According to the CPAK classification, males predominantly exhibited CPAK type I (38.8%) and type II (27.3%), whereas females displayed a more evenly distributed pattern across CPAK type I (22.7%), type II (27.3%), and type III (25.7%) (*p* < 0.001) [21]. In our cohort of arthritic patients, similar trends were observed, with females demonstrating a mean aHKA of −2.02 ± 5.31 degrees, indicative of a neutral alignment within acceptable boundaries, while males exhibited a mean aHKA of −4.28 ± 4.51 degrees, corresponding to a varus alignment. Regarding CPAK categorization, type I (39.2%) and type II (18.67%) were most prevalent among females, whereas type I (52%) predominated in males, with Type IV ranking as the second most common subtype (18.4%). These findings demonstrate a considerable degree of alignment with previously documented research, underscoring the consistency observed across various studies in the field.

Various methodologies for aligning implants in TKA surgery have been developed, encompassing mechanical, kinematic, and functional approaches. Each alignment philosophy ultimately aims to achieve the same goal: enabling pain-free patient ambulation. Mechanical alignment, a widely employed technique since the inception of TKA, seeks to establish a neutral mechanical axis of the limb. However, this approach has sparked considerable debate, as only a small percentage of the population exhibits a naturally neutral knee alignment [22,23,24]. MA aims for CPAK type V alignment, characterized by a neutral alignment and a joint parallel to the ground. Our study findings revealed that only a minority of subjects possess this phenotype, comprising 7.4% in the osteoarthritic group and 9.6% in the healthy group. It is noteworthy that the most prevalent phenotype in the arthritic group is type I (42.4%), suggesting that transitioning from a varus alignment to a neutral one may not necessarily yield significant benefits for the patient, potentially impacting soft tissue balance. Studies conducted on arthritic populations have revealed variability in the prevalence of CPAK type V across different countries: Portugal (15.0%), USA (12.5%), Turkey (12.3%), Japan (4.4%), Korea (19.8%), and India (11.6%). However, none of these studies reported a predominance of this phenotype [7,18,19,25,26,27].

Achieving neutral mechanical alignment is widely regarded as essential for the success and longevity of knee arthroplasty procedures. The prevailing consensus suggests that neutral alignment facilitates balanced mediolateral joint loading, thus promoting durability and success in arthroplasty outcomes. MacDessi et al. conducted a study examining intracompartmental pressure (ICP) in a cohort of 125 patients undergoing primary total TKA to assess the proportion of balanced knees at 10 degrees of flexion for each CPAK type using KA versus MA techniques. Their investigation revealed a notable difference in ICP among CPAK types I, II, and IV, with MA demonstrating a more pronounced disparity compared to KA. These findings underscore the imperative of adopting a personalized approach for patients, as MA may not be universally optimal for all individuals undergoing TKA. The study emphasizes the utility of CPAK classification in discerning patients who stand to derive the greatest advantage from a kinematic alignment implant approach [7,12].

Recent studies have investigated intra- and inter-observer correlation using CPAK classification to assess the reliability and consistency of knee alignment phenotype categorization. Intra-observer correlation examines the agreement between multiple assessments made by the same observer, while inter-observer correlation assesses agreement among different observers. A study by Mulpur et al. evaluated intra and inter-observer correlation among orthopedic surgeons using CPAK classification assessed in both groups (OA and healthy). The researchers found high correlation coefficients (>0.9), indicating strong agreement within individual observers across multiple assessments [28]. Similarly, a study by Senel et al. obtained high intra- and inter-observer correlations based on LDFA and MPTA measurements (>0.9) [17]. In our study, both the intra- and the interobserver agreement were excellent, suggesting that the CPAK classification demonstrates reliability and consistency in clinical practice. However, ongoing efforts to standardize training protocols, refine classification criteria, and ensure proficiency among observers remain crucial for optimizing the utility of CPAK classification in the assessment of knee alignment.

Corbett et al. conducted a retrospective computed tomographic study to investigate the alignment across various types of CPAK phenotypes in a cohort of patients who underwent robotic-assisted primary TKA. The study aimed to evaluate coronal, sagittal, and rotational alignment and to determine the necessity of extending the classification beyond the coronal plane. Data from 437 patients who underwent the procedure were analyzed. The results revealed no significant differences in tibial slope or femoral rotation among CPAK phenotypes, except for type III knees, which exhibited greater tibiofemoral rotation compared to types I, II, IV, and V. In conclusion, the study suggests that there is no compelling reason to expand the CPAK classification beyond the assessment of coronal plane alignment [29].

Robot-assisted total knee arthroplasty has emerged as a pivotal domain in contemporary orthopedics, aiming to mitigate human error and enhance patient satisfaction rates and plays a crucial role in achieving precise coronal plane alignment of the knee. By utilizing advanced robotic systems, surgeons can meticulously plan and execute implant placement according to preoperative plans, which often include target-based parameters such as the aHKA and JLO. The CPAK classification serves as a valuable adjunct tool in meticulous preoperative planning. When integrated with robotic technology, it facilitates improved surgical precision and ultimately augments patient outcomes [30,31,32].

Limitations inherent in this study include potential radiological measurement errors stemming from rotational deformities of the distal femur and malalignment of the knee attributable to advanced knee osteoarthritis. Furthermore, an uneven distribution of participants among the groups poses another limitation, with a predominance of females in the arthritic group and males in the healthy group. Although this study is conducted at a single center, our patient cohort is drawn from various regions across the country, owing to our monodisciplinary orthopedic profile. Therefore, we contend that our findings may hold relevance to our specific geographic area.

## 5. Conclusions

In summary, our investigation using the CPAK classification in the Romanian population highlighted notable trends in knee phenotypes among healthy and arthritic individuals. We observed CPAK type I as predominant in the arthritic cohort and type II in the healthy population, consistent with previous research across diverse populations. Moreover, our study aligns with existing research demonstrating gender-specific patterns. The robust intra- and inter-observer correlation observed in our study, mirroring findings from recent research, serves to reinforce the reliability and consistency of the CPAK classification within clinical practice. Ultimately, our findings suggest that comprehending knee phenotypes is instrumental in improving functional outcomes and enhancing patient satisfaction rates following TKA. CPAK classification demonstrates its significance in achieving these goals.

## Figures and Tables

**Table 1:** CPAK classification into 9 phenotypes [12].

	Varus	Neutral	Valgus
**Apex distal**	**CPAK I**	**CPAK II**	**CPAK III**
	aHKA < −2°	aHKA 0° ± 2°	aHKA > 2°
	JLO < 177°	JLO < 177°	JLO < 177°
**Neutral**	**CPAK IV**	**CPAK V**	**CPAK VI**
	aHKA < −2°	aHKA 0° ± 2°	aHKA > 2°
	JLO 180° ± 3°	JLO 180° ± 3°	JLO 180° ± 3°
**Apex proximal**	**CPAK VII**	**CPAK VIII**	**CPAK IX**
	aHKA < −2°	aHKA 0° ± 2°	aHKA > 2°
	JLO > 183°	JLO > 183°	JLO > 183°

aHKA = arithmetic hip knee ankle angle; JLO = joint line obliquity. Adapted with permission from [12].

**Table 2:** Demographic characteristics of the study population and differences between groups.

Parameter	Osteoarthritic (*n* = 500)	Healthy (*n* = 500)	*p* Value
Mean age (years)	67.99 ± 7.18 [67.36–68.62]	35.99 ± 14.21 [34.74–37.24]	<0.001
Male	25.40% (*n* = 125)	66.00% (*n* = 330)	<0.0001
Female	74.60% (*n* = 375)	34.00% (*n* = 170)	
Mean BMI (kg/m^2^)	30.75 ± 3.95 [30.39–31.11]	26.49 ± 4.15 [26.12–26.86]	<0.0001
Mean mLDFA (degrees)	88.78 ± 3.23 [88.49–89.06]	87.25 ± 2.20 [87.06–87.44]	<0.0001
Mean mMPTA (degrees)	86.19 ± 3.41 [85.89–86.49]	87.07 ± 2.31 [86.87–87.27]	<0.0001
Mean JLO (degrees)	174.97 ± 4.12 [174.61–175.33]	174.32 ± 3.24 [174.04–174.61]	0.0059
Mean aHKA (degrees)	−2.59 ± 5.21 [−3.04–−2.13]	−0.18 ± 3.13 [−0.46–0.09]	<0.0001
Kellgren–Lawrence score			<0.0001
0	-	44.6% (*n* = 223)	
1	-	55.4% (*n* = 277)	
2	13.2% (*n* = 66)	-	
3	23.4% (*n* = 117)	-	
4	63.4% (*n* = 317)	-	

95% confidence intervals provided in the brackets.

**Table 3:** CPAK type distribution according to MacDessi classification [12].

CPAK Type	Cohort 1, Osteoarthritic (*n* = 500)			Cohort 2, Healthy (*n* = 500)		
	Male (*n*, %)	Female (*n*, %)	Total (*n*, %)	Male (*n*, %)	Female (*n*, %)	Total (*n*, %)
I	65, 52.00%	147, 39.20%	212, 42.40%	95, 28.79%	17, 10.00%	112, 22.40%
II	14, 11.20%	70, 18.67%	84, 16.80%	139, 42.12%	56, 32.94%	195, 39.00%
III	8, 6.40%	41, 10.93%	49, 9.80%	45, 13.64%	48, 28.24%	93, 18.60%
IV	23, 18.40%	58, 15.47%	81, 16.20%	16, 4.85%	9, 5.29%	25, 5.00%
V	8, 6.40%	29, 7.73%	37, 7.40%	21, 6.36%	27, 15.88%	48, 9.60%
VI	2, 1.60%	21, 5.60%	23, 4.60%	10, 3.03%	12, 7.06%	22, 4.40%
VII	2, 1.60%	4, 1.07%	6, 1.20%	2, 0.61%	1, 0.59%	3, 0.60%
VIII	1, 0.80%	4, 1.07%	5, 1.00%	1, 0.30%	0, 0.00%	1, 0.20%
IX	2, 1.60%	1, 0.27%	3, 0.60%	1, 0.30%	0, 0.00%	1, 0.20%

## Data Availability

The datasets used and/or analyzed during the current study are available from the corresponding author upon reasonable request.
